# Mind-Mindedness and Parenting Stress: A Cross-Sectional Study in a Cohort of Mothers of 3-Month-Old Full-Term and Preterm Infants

**DOI:** 10.3390/ijerph17217735

**Published:** 2020-10-22

**Authors:** Chiara Suttora, Maria Spinelli, Tiziana Aureli, Mirco Fasolo, Francesca Lionetti, Odoardo Picciolini, Maura Ravasi, Nicoletta Salerni

**Affiliations:** 1 Department of Psychology “Renzo Canestrari”, University of Bologna, 40127 Bologna, Italy; 2 Department of Neurosciences, Imaging and Clinical Sciences, University G. D’Annunzio Chieti-Pescara, 66100 Chieti, Italy; maria.spinelli@unich.it (M.S.); t.aureli@unich.it (T.A.); mirco.fasolo@unich.it (M.F.); francesca.lionetti@unich.it (F.L.); 3 Fondazione IRCCS Ca’ Granda Ospedale Maggiore Policlinico, Pediatric Physical Medicine & Rehabilitation Unit, 20122 Milan, Italy; odoardo.picciolini@policlinico.mi.it; 4 Fondazione IRCCS Ca’ Granda Ospedale Maggiore Policlinico, NICU, 20122 Milan, Italy; maura.ravasi@gmail.com; 5 Department of Psychology, University of Milano-Bicocca, 20126 Milan, Italy; nicoletta.salerni@unimib.it

**Keywords:** preterm birth, mind-mindedness, parenting stress, mother–infant interaction, parenting

## Abstract

The preterm birth of a child is a sudden event that can disturb the overall family system and its functioning. Many studies have been conducted with the aim of exploring how and the degree to which this event affects the early mother–infant dyadic relationship and maternal well-being, with often mixed findings. The present study investigates the combined effect of preterm birth and parenting stress on mind-mindedness, a parenting dimension that captures how parents represent and treat their children as separate individuals with their own mental states and activities. A hundred and ten mothers and their three-month-old infants (preterm = 54; full-term = 56) participated in the study. Mind-mindedness was assessed by coding mothers’ comments about infant’s mental states during dyadic face-to-face interaction. Parenting stress was evaluated with the Parenting Stress Index Short Form questionnaire. Mothers of preterm infants reported similar levels of appropriate and non-attuned mind-related comments to mothers of full-term infants. The reported parenting stress levels were also comparable. Interestingly, only mothers of preterm infants who reported higher stress in parenting showed more non-attuned comments during the interaction. The results underline the need to address preterm birth as a complex event, going beyond group differences and considering its interplay with other risk or protective factors in shaping children’s and parents’ adjustments and well-being.

## 1. Introduction

The unexpected end of a pregnancy that culminates in a preterm (PT) birth represents a risk for the infant but also for the mother and the dyad. Many PT infants manifest interactive difficulties, and their mothers seem to be less sensitive to infants’ needs compared to full-term (FT) infants’ mothers [1]. The mothers of PT infants report obstacles in their transition to parenthood, with a detrimental representation of themselves as good and effective mothers and difficulties in seeing the infant as healthy and easy to care for [2,3,4].

The present study explores how the perceived stress associated with parenting a three-month-old infant interacts with PT birth in affecting a behavioral dimension of parenting that is a direct measure of how the mother is able to represent, understand, and comment on infant mental needs—her mind-mindedness [5].

### 1.1. Preterm Birth and the Mother–Infant Dyad

According to the World Health Organization [6], an infant is preterm when she/he is born before completing the 37th week of gestation. PT birth constitutes an exceptional condition that could affect several areas of the newborn’s development, with difficulties increasing as a function of the severity of prematurity [7]. PT infants often display early developmental delays and interactive and communicative difficulties [8,9], such as diminished alertness and responsivity [10,11,12], greater passivity [13], and fewer expressions of positive affect [14]. Associated with these interactive difficulties, PT infants may also manifest temperamental difficulties, including increased irritability, fussiness, and inconsolability compared to their FT counterparts [15,16]. Mothers may be affected by PT birth as well [17]. The neonatal intensive care unit (NICU) experience may expose mothers to high levels of psychological distress and post-traumatic stress [18,19,20,21,22,23,24], as the infants are often surrounded by tubes and medical equipment during their recovery. This may lead mothers to perceive and represent them as too fragile, less competent, and more difficult to care for [4,25].

PT infants’ characteristics and specific difficulties together with the not-normative parental experience are thought to affect parental well-being and interactive behaviors. This hypothesis has been widely addressed by the literature on PT birth, but the findings, all in all, have failed in providing a clear picture of such an extremely complex phenomenon.

A substantial amount of research has focused on how raising a PT infant is a potentially stressful and overly demanding experience, with studies yielding controversial findings [26,27,28,29,30,31,32]. A recent meta-analysis shed some light on these mixed findings, concluding that the parents of PT children experience only slightly more stress than the parents of FT children, with very small effect sizes [33]. The authors suggested that some PT infants’ parents may perceive parenting as more stressful than others due to their infants’ multiple health conditions [34].

With respect to mothers’ interactive behavior, a large number of studies have reported difficulties for mothers interacting with their PT infants [18]. Investigations from the third month of the infants’ life onward showed that mothers of PT infants are less emotionally involved, less contingent, and less congruent with infants’ signals [35], showing a lower maternal sensitivity [36,37]. Other works have documented that not all the mothers show the same difficulties, reporting high levels of intrusiveness only in extremely low birth weight infants’ mothers, indicating that it is only when mothers have to deal with more severe PT infants that interactive difficulties are present [38]. Other studies have failed to find differences among mothers with respect to sensitivity [39], with a recent meta-analysis observing a general lack of evidence of differences in the sensitivity of mothers of PT and FT infants [40].

Similar results were found by recent studies addressing the effect of PT birth on maternal mind-mindedness [41,42], a behavior that represents an index of maternal ability to mentalize and reflect infant mental states, “a form of mentalization in action” [43]. More than other constructs, mind-mindedness is influenced by the complexity of the representations that mothers develop of their infants because, by definition, it consists of the maternal tendency to consider the infant as a subject characterized by different and changeable mental states [5]. Although we may expect that the disrupted representations of PT infants, who can be perceived as too fragile, less competent, and more difficult to care for, would interfere with the mothers’ ability to view their infants as mental agents and then negatively affect their maternal mind-mindedness, empirical studies have found no direct effect of PT birth on the quality of mind-mindedness [41,42]. 

To sum up, even if PT birth may represent a critical condition affecting dyadic well-being at different levels, divergent findings are present with respect to mothers’ perception of parenting a PT infant as a stressful experience [26,27,28,29,30,31,32,33] and mothers’ interactive difficulties, as the use of mind-related comments during the interaction, especially during the first months of an infants’ life [18,38,39,41,42,44]. The hypothesis guiding the present study is that PT birth is a complex phenomenon resulting from the interaction of the infant and maternal characteristics and experiences. When addressing the effects of PT birth on the quality of parenting, we should go beyond group differences and examine the interaction among different factors [19,34,45]. In this regard, since high levels of parenting stress may negatively affect the quality of maternal interactive behavior—with more stressed mothers showing less positive affect, less involvement, and fewer verbalizations [46]—and since prematurity constitutes a particular risk condition, we may expect to find that only more stressed PT mothers, and not all of them, display a lower quality of parenting.

### 1.2. Study Aims and Hypotheses

The current study aims to explore the role of PT birth and the perception of parenting as a stressful experience in maternal mind-mindedness during free-play mother–infant interactions with infants at three months of age. The choice of investigating these relationships at this early age regards both theoretical and practical reasons. Firstly, this is a particularly relevant period for the development of dyadic interaction patterns and co-regulatory processes; secondly, the early identification of difficulties in maternal mind-mindedness can aid in the implementation of effective and appropriately timed clinical protocols and interventions.

Since this is the first study to address the topic from this perspective, we consider it explorative. In this sense, the study focuses on a group of mothers of low-risk PT infants heterogenous for gestational age and birthweight and a group of mothers of FT infants.

Based on the literature discussed above, the study aims and expected results are the following:

1. To assess the role of preterm birth condition in maternal mind-mindedness and parenting stress. No differences between mothers of preterm and full-term infants are expected regarding the use of appropriate and non-attuned mind-related comments and in the reported levels of parenting stress.
2. To investigate the relationship between parenting stress and maternal mind-mindedness. We expect that higher levels of parenting stress will be related to lower maternal mind-mindedness (i.e., a low use of appropriate mind-related comments and a high use of non-attuned mind-related comments), with a stronger effect in the PT group.
3. To examine the interaction between birth condition and parenting stress on maternal appropriate and non-attuned mind-mindedness. We hypothesize that the interaction between PT birth and high levels of parenting stress will negatively affect the quality of maternal mind-mindedness.

## 2. Materials and Methods

### 2.1. Study Design and Participants

The present study is a cross-sectional study framed in a longitudinal cohort project aimed at investigating the role of preterm birth in the development of mother–infant interactions during the first year of life. The study here presented includes data from the first time point of the cohort project exclusively. The invited participants were 57 PT and 60 FT infants aged three months (corrected age for PT infants) and their mothers. The inclusion criteria for PT infants were having a gestational age between 25 and 37 weeks at birth. The exclusion criteria for the PT and FT groups were the presence of genetic abnormalities, severe neurofunctional impairments, severe neonatal complications and/or neurosensory disabilities (i.e., blindness or deafness), and not having signed the informed consent. Mothers of PT infants eligible for the study were invited by nurses and medical doctors to participate in the study in the postnatal follow-up services of the Neonatal Intensive Care Unit of three main hospitals in northern and central Italy. The mothers of FT infants were contacted in the Maternal and Infant Health Care Services of the same hospitals after delivery. The hospitals agreed to participate in the project due to consolidated research collaborations with the scholars involved in the present study. The PT and FT infants were equally distributed among the three hospitals and were invited to participate in the study over a 1-year data collection period, with the recruitment of participants starting in the year 2017 and ending by January 2019. Seven mothers—three in the PT group and four in the FT group—did not return the requested questionnaires, so they were excluded from the following data analyses. The final sample size includes 110 participants: 54 PT and 56 FT.

Both parents signed consensus forms for study participation. All the procedures performed in studies involving human participants were in accordance with the ethical standards of the institutional and/or national research committee (ethic committee of the University of Milano Bicocca, n°231) and with the 1964 Helsinki Declaration and its later amendments or comparable ethical standards.

### 2.2. Procedure and Measures

At three months of infant age (PT: *M* = 3.02, *SD* = 0.04; FT: *M* = 3.01, *SD* = 0.04), the mothers were invited to participate in the data collection that was the focus of the present study. The data collection consisted of the administration of questionnaires and the video-recording of the mother–infant dyadic interaction and lasted around 20 min. The video-recording session consisted of a three-minute face-to-face interaction with the infant seated in a baby seat and the mother placed in front of him or her facing a mirror located behind the infant’s seat so that both partners’ faces could be clearly seen. The interactions were carried out with similar settings in an appropriate room of each hospital for PT dyads and in the Developmental Psychology Lab of two universities for FT dyads. The mothers were asked to interact with their infants as they would if they were at home. The length of the session corresponded to what was suggested in the 2.2 version of the Mind-mindedness Coding Manual [47] for mind-mindedness coding in mothers of very young infants. At the end of the video-recording session, the mothers were also asked to complete and return in person or by e-mail the Parenting Stress Index questionnaire and a socio-demographic form including maternal and infant information.

#### 2.2.1. Mind-Mindedness

The mind-mindedness coding system [47] was developed by Meins and collaborators [5,48] and stems from attachment theory, tapping into caregivers’ representation of children’s mental life. Differently from other tools for investigating parents’ representations of the infant [49,50], observational mind-mindedness is not measured by the assessment of the quality, structure, and contents of the subjective parental narrative about the child. Rather, in the first year of life, mind-mindedness is measured as the caregiver’s tendency to comment—appropriately or not [51,52]—on the infant’s putative mental states during social interaction, through the transcription and analysis of parental verbal comments. During the interaction with the infant, parents can refer to the infant’s mental states appropriately: this is the case when parental comments appear able to reflect what the infant is likely to be feeling or thinking in that specific moment. However, parents can also use a mental state lexicon to address their infants in a non-attuned manner when, for instance, the state captured is a misinterpretation of the infant’s real inner condition not linked to an observable behavior or attitude of the infant. The literature indicates that, while the former type of mind-related comments (appropriate mind-related comments) partially reflects the conventional notion of parental sensitivity and responsivity, the latter (non-attuned mind-related comments) could indicate a caregiver’s lack of attunement to the infant’s internal state and/or, in some cases, the imposition of caregivers’ own thoughts and emotions [51,53]. Several studies have confirmed that maternal mind-mindedness, measured with this observational approach, is a stable dimension that is highly consistent across relationships and with a good level of reliability [52,54,55,56].

Therefore, from the videotaped sessions, the maternal verbal input directed to the infant was fully transcribed, and maternal verbal comments were coded following the Mind-mindedness Coding Manual, version 2.2 [47]. Each maternal comment including a verbal reference to the infant’s mental state—for instance, a reference to her preference for something (such as a toy), desires, cognitions, emotions, attempts to manipulate others’ beliefs, epistemic states, and intentions—was coded as mind-related. Additionally in this category were verbal comments in which mothers talked for the infant (e.g., “I really love this song, Mommy!”), as in this type of verbal comment the mothers used words that described the infant’s inner state. Once a comment was coded as mind-related, the coder further classified it as an appropriate mind-related comment (AMRC) or a non-attuned mind-related comment (NAMRC) according to the following criteria retrieved from the coding manual. A mind-related comment was considered appropriate (a) when the coder agreed on the maternal interpretation of the child’s current mental state based on the child’s observable behavior (e.g., the mother says “You’re really happy today” when the baby is smiling); (b) when the comment links the current activity with similar events in the past or future (e.g., the mother says “you remember playing with your sister’s giraffe, don’t you?” while playing with a giraffe toy); (c) when the mother uses a comment to clarify how to proceed after an interactive pause (e.g., the mother says “do you want to sing a song now?”). Mind-related comments were coded as non-attuned when the mother failed to identify the child’s current internal state correctly, either by misinterpreting the infant’s state or attributing to her a mental activity in the absence of observable behavioral cues (e.g., the mother says “I know, you miss dad” when the infant is not showing any sign of distress or sadness, or the mother says “You really love this rattle” when the infant is showing no interest in the toy).

In the present study, the number of appropriate mind-related comments (AMRC) and the number of non-attuned mind-related comments (NAMRC) were used as measures of maternal mind-mindedness. As suggested by Meins and Fernyhough [47], to control for maternal verbosity the proportional frequencies of AMRC and NAMRC comments in relation to the total number of maternal verbal utterances transcribed were computed.

Two independent observers coded the sessions. Twenty percent of the sessions were selected randomly to test reliability, then coded by both observers. The interrater reliability between the two coders, tested with intraclass correlation, was 0.98 for appropriate comments and 0.86 for non-attuned comments.

#### 2.2.2. Parenting Stress

For the study’s aims, the mothers were asked to complete an online version of the Parenting Stress Index Short Form (PSI–SF) questionnaire [57,58]. The PSI–SF is a questionnaire commonly used to measure stress in the parent–child system and to identify those caregivers most in need of support. The PSI–SF includes 36 items rated from 1 to 5 on a Likert scale (1 = *strongly disagree*; 5 = *strongly agree*) and consists of three subscales, each with 12 items: Parental Distress (PD), Parent–Child Dysfunctional Interaction (P–CDI), and Difficult Child (DC). High values indicate more parenting stress. The Parental Distress subscale explores the stress related to the parent’s perception of her/his child-rearing competences, the level of spousal conflict or support, and the restrictions placed on him or her by the parental role. The Parent–Child Dysfunctional Interaction subscale refers to a parent’s perception of the difficulties in the parent–infant interaction. The Difficult Child subscale items concern the parent’s view of the difficulty of the child’s temperament and demandingness. As some PSI items were unanswered (the maximum number of missing items being 2 for each subject), the subscales and total scale scores were expressed as means. The use of mean scores also allows an easy comparison among PSI subscales. The internal consistency of the current sample was satisfactory and equal to Cronbach’s *α* 0.86 for PD, 0.86 for P–CDI, and 0.90 for DC.

#### 2.2.3. Sociodemographic Questionnaire

A socio-demographic questionnaire was developed ad hoc for the study’s purposes. The participants were asked to provide information on maternal age, education, and civil status (defined as living with partner or not), as well as on the infant’s birth-order (firstborn vs. laterborn), the presence of siblings (only child vs. with siblings), and the gestational age and the weight at birth.

## 3. Data Analysis

Analyses were run with IBM-SPSS version 23 (IBM Corp., Armonk, N.Y., USA) and R software (R Core Team, Vienna, Austria). All the tests were bilateral and the level of statistical significance was set at 0.05. First, the distribution of data was assessed by calculating the variables of skewness and kurtosis. The values for the PSI subscales and AMRC were within reasonable limits, with the skewness ranging from −1.32 to 0.69 and the kurtosis from 0.11 to 1.47. Data for the NAMRC variable were highly skewed (2.29) and high in kurtosis (6.12). The Kolmogorov–Smirnov test indicated that all the variables were not normally distributed (*ps* < 0.05). Because the data were skewed, we ran the nonparametric tests the Mann–Whitney U and χ^2^ for preliminary analyses and the nonparametric Spearman’s ρ to test the second hypothesis, and to explore the first and third hypothesis we used the Generalized addicted model for location, scale, and shape (GAMLSS) [59]. The latter specifically enables flexible analysis with not normally distributed data.

The first preliminary analyses (Mann–Whitney U and χ^2^) explored if differences between groups were present according to the characteristics of the sample.

To test the first hypothesis, we carried out a set of analyses (GAMLSS) directed at exploring differences in the use of mind-mindedness comments, both appropriate and non-attuned, and in levels of parenting stress, as assessed with the PSI-SF, in mothers of PT and FT infants.

To test the second hypothesis, a set of Spearman’s ρ correlations were used to explore the relationships between mothers’ reported parenting stress scores and their use of appropriate and non-attuned mind-related comments, first taking into account all mothers and, finally, considering the two groups separately.

Finally, to test the third hypothesis, the moderating effects of parenting stress on the relationship between birth status and the use of mind-mindedness comments were tested to verify the presence or absence of interaction between the stress of parenting and birth status on maternal appropriate and non-attuned use of mind-related comments. Moderation analyses were performed with the GAMLSS package [59] using the zero-inflated beta distribution.

## 4. Results

### 4.1. Characteristics of the Study Sample

Descriptive statistics for the mothers’ and infants’ characteristics are reported in Table 1, together with the results of the between-group comparisons.

The mothers of PT and FT infants were similar in terms of age but differed regarding their educational level, with mothers of PT infants showing, on average, fewer years of education than the other mothers. The mothers were all living with a partner, except for one PT infant’s mother. The infants in the PT and FT groups were comparable for gender, birth order, and the presence of siblings, with the majority of the infants recruited being firstborn and only children. The PT infants and FT infants had a mean gestational age of 31.03 and 39.48 weeks, respectively, and a mean birth weight of 1351 and 3342 g, respectively. The PT infants were hospitalized on average for 44.78 days (*SD =* 21.69).

### 4.2. Mind-Mindedness and Parenting Stress in Mothers of Preterm and Full-Term Infants

Table 2 shows the descriptive statistics of measures of maternal mind-mindedness and PSI scores for the mothers of PT and FT infants and for all the mothers involved in the study. Between-group comparisons on the use of appropriate and non-attuned mind-related comments and on levels of parenting stress were performed with a set of GAMLSS controlling for the effects of maternal educational levels, which differed significantly between groups. The results indicated the absence of significant differences due to birth condition in measures of maternal mind-mindedness, with the covariate maternal education resulting in being not significant in any of the models tested (all *ps* = ns). On average, 11% of the maternal comments referred to infants’ mental state, with 9% of the comments being appropriate for the infant’s ongoing mental activity and the remaining 2% being non-attuned to the infants’ inner state. As for parenting stress, the results showed that the mothers of PT and FT infants reported comparable levels of stress in all the PSI domains.

### 4.3. Relationship between Parenting Stress Levels and Maternal Mind-Mindedness

In the following analyses, the relationships among the study’s variables were first investigated on the whole sample of participants, independent of the infant’s birth condition (Table 3).

Appropriate and non-attuned mind-related comments were completely independent, showing correlation values close to zero. Regarding parenting stress, the results showed high and significant correlations among PSI subscales, indicating that the stress levels reported in the parent, child, and dyadic interaction domains were positively related. Regarding the relationship between parenting stress and maternal mind-mindedness, the results indicated that a greater use of non-attuned mind-related comments was related with higher reported stress in the PSI Difficult Child domain. To examine this result further, correlation analyses were conducted separately for the FT and PT groups. Table 4 shows that the PSI Difficult Child scores were positively related to the use of non-attuned mind-mindedness comments only in the mothers of PT infants.

### 4.4. Moderation Effects of Parenting Stress on the Relationship between Preterm Birth and Mind-Mindedness

To test the moderating effects of child-related parenting stress, as measured by the PSI Difficult Child subscale, on the relationship between birth condition and mind-mindedness, two moderation models were hypothesized and tested with the GAMLSS. The first tested how stress on the PSI Difficult Child subscale moderated the relationship between birth condition (PT vs. FT) and the use of appropriate mind-related comments (AMRC). The second model tested the same effect on the use of non-attuned mind-related comments (NAMRC). Neither model yielded significant results for interactions, thus implying the absence of significant moderation effects of child-related parenting stress (PSI Difficult Child) on the relationship between birth condition and appropriate (PSI DC X birth condition on AMRC: *B* = −0.14, *SE* = 0.30, *p* = 0.640) and not-attuned dimensions of maternal mind-mindedness (PSI DC X birth condition on NAMRC: *B* = 0.21, *SE* = 0.43, *p* = 0.633). Considering these findings, the hypothesis of an interaction between birth status and parenting stress, with high levels of parenting stress and PT birth negatively affecting the quality of maternal mind-mindedness, was rejected. 

## 5. Discussion

The present study aimed to shed light on the role of PT birth and parenting stress on maternal mind-mindedness, an interactive behavior considered an index of maternal ability to represent and treat the child as a mental agent, which fosters children’s emotional and cognitive development [48,60]. Our guiding hypothesis was that PT birth could not constitute a relevant challenge for parents trying to interpret infants’ psychological needs and states per se, but only when they perceive their parenting role and experience as stressful. The study’s main findings contributed to clarifying this phenomenon by evidencing, only in the PT group, a relationship between parenting stress related to the child and the appropriateness of maternal mind-related comments during the interaction with the infant.

### 5.1. Effects of Preterm Birth on Parenting Stress and Mind-Mindedness

The findings supported our first hypothesis; PT birth condition itself yielded no significant effects on either parenting stress or maternal mind-mindedness. In terms of parenting stress, the results are in line with the conclusions of the more recent literature that failed to find differences between the groups [29,30], especially in low-risk samples, such as the one included in our study [33].

Similarly, the lack of interference of PT birth per se with the mothers’ ability to view their infants as mental agents corroborates the literature reporting the limited role played by PT birth alone in caregivers’ mind-mindedness [41,42] and related parenting abilities, such as sensitivity, responsiveness, and reflective functioning [38,40,61,62].

As previous studies evidenced, the characteristics of PT infants may make a difference in this process, with more at-risk samples being more affected [38]. Our study evidenced that, in low-risk PT infants’ mothers, this relevant aspect of dyadic interaction is not impaired.

### 5.2. Preterm Birth and Parenting Stress on Mind-Mindedness: A Combined Effect?

The second set of analyses gave a novel contribution to the literature, confirming our second hypothesis regarding stronger effects of parenting stress on mind-mindedness in the PT sample. We not only found a relationship between parenting stress and the quality of maternal mind-mindedness in the whole sample, as previously evidenced by other studies [63,64], but also, when considering the groups separately, higher levels of parenting stress in the Difficult Child domain resulted in a greater use of non-attuned mind-related comments only in PT infants mothers. The Difficult Child subscale investigates how parents perceive their infants as stressful to manage; for instance, parents are asked to rate whether their infants are fussier and more difficult to regulate than others. Mothers in the PT group that perceived their infants as particularly difficult tried to get in touch with their infants‘ mental states and activities, probably in order to calm and regulate them. However, they did so in a not-attuned manner, trying to find a correct understanding of their infants but unsuccessfully. Based on the previous literature, we can speculate that this breakdown in the mentalization ability of the mothers of PT infants reporting high parenting stress can be related to these infants’ lack of responsivity and passivity, characteristics that can make them less easy to read [15]. These findings underscore how meaningful it is to understand how parenting stress relates to maternal behaviors and attitudes in the mothers of PT infants. This is even more true considering that sensitive parenting represents an important protective factor from negative outcomes, especially for vulnerable PT children [65,66].

Even if the correlations suggested the need for an exploration of the moderation effect, we failed to find support for our third hypothesis. Parenting stress did not interact with PT birth in interfering with the ability to read the infant’s intentions, preferences, emotions and cognitions appropriately. A very recent study examining mind-mindedness predictive factors in PT mothers evidenced the mediating role of environment-driven stress sources, with poorly organized families faced with PT birth—a condition that requires extra effort to rethink the family system and organization—showing fever mind-related comments [42]. This may suggest that considering only a perceived condition of distress related to parenting, like the one we explored, is not enough to fully understand the complexity of the stressful experience these parents may have and its subsequent effects on parenting.

### 5.3. Limits and Implications for Future Studies

Some limits of the present study deserve to be mentioned. Firstly, PT infants are a wide and variable population and considering them as a whole group is a limitation. Our sample included mainly low-risk PT infants and healthy mothers, and this did not allow us to explore the role of other infant and maternal individual risk factors, such as the medical condition of both of them, which may make parenting even more challenging. However, our results evidenced that, even in a low-risk sample, some critical aspects of the maternal ability to understand and refer to infants’ mental states may be evidenced and deserve to be explored. Secondly, the role of other environmental and socio-economic factors that were not considered by the present study may affect the quality of parental adjustment, as well as other maternal risk factors that may have also influenced the PT delivery. Finally, we did not consider the role of maternal representations of herself as a mother; the traumatic experience of PT birth; and other behavioral aspects of maternal interaction such as emotional expressions, touch, and the prosody of the voice. The inclusion of these aspects can help us to enrich the understanding of the complex phenomenon of mother–preterm infant interaction. 

Future studies should explore this phenomenon longitudinally, taking into consideration the role of other individual and dyadic factors as predictors of maternal mind-mindedness ability. Our sample has been collected in only one country even if in different cities, so to generalize the results further explorations in other cultures may be necessary as well as analyses in more at-risk samples.

## 6. Conclusions

This is the first study to explore both the perception of parenting as stressful and maternal mind-mindedness in a sample of PT and FT dyads. Our results contributed to the literature by suggesting that being a mother of a PT infant is not per se a risk condition. It is when mothers of PT infants report high stress in parenting their infants that the quality of mind-mindedness may be impaired.

The conclusion drawn from the study’s findings has relevant clinical implications. In order to fully understand how to prevent PT infants’ and their mothers’ negative outcomes, PT birth must be considered as a complex experience with multifaced implications for parenting. Thus, parenting stress should be considered in the follow-up routines as a crucial risk factor, especially for PT mother–infant dyads. Furthermore, since maternal mind-mindedness is crucial for child development, interventions focused on improving this aspect of maternal interaction should be promoted to prevent future delays in infants and children [67,68].

## Figures and Tables

**Table 1:** Descriptive data and comparisons between groups.

Socio-Demographic Measures	PT (*n* = 54)	FT (*n* = 56)	*p*
Maternal Age (years), *M* (*SD*)	34.20 (4.71)	35.05 (4.12)	0.393
Maternal Education (years), *M* (*SD*)	15.04 (3.30)	16.75 (2.38)	0.003
Living with partner, *n* (%)	53 (98.14)	55 (100)	0.306
Male gender, *n* (%)	24 (44.44)	29 (51.78)	0.441
Firstborn, *n* (%)	46 (85.18)	43 (76.78)	0.262
Only-child, *n* (%)	38 (70.37)	40 (71.42)	0.903
GA (weeks), *M* (*SD*)	31.03 (2.36)	39.48 (1.17)	<0.001
BW (grams), *M* (*SD*)	1351 (335.27)	3342 (431.66)	<0.001

FT = Full-Term; PT = Preterm; BW = Birth Weight; GA = Gestational Age. χ^2^ test was used to compare groups regarding maternal civil status, child gender, birth order, and the presence of siblings; the Mann–Whitney U test was used for maternal age and education and the infant’s GA and BW.

**Table 2:** Descriptive statistics and results of GAMLSS on the measures of mind-mindedness and parenting stress in mothers of preterm and full-term infants.

Maternal Variables	*N*	PT	FT	Total	*B*	*SE*	*p*
		*M* (*SD*)	*M* (*SD*)	*M* (*SD*)			
Appropriate mind-related comments	110	0.09 (0.06)	0.10 (0.07)	0.09 (0.07)	−0.05	0.13	0.685
Non-attuned mind-related comments	110	0.01 (0.02)	0.02 (0.03)	0.02 (0.03)	−0.02	0.19	0.895
PSI—Parental Distress	109	1.90 (0.64)	2.01 (0.61)	1.95 (0.62)	−0.07	0.12	0.572
PSI—Child Difficult Interaction	110	1.45 (0.45)	1.46 (0.36)	1.45 (0.40)	−0.03	0.08	0.670
PSI—Difficult Child	110	1.63 (0.42)	1.58 (0.43)	1.60 (0.43)	0.01	0.08	0.989
PSI—Total Stress	110	1.66 (0.41)	1.68 (0.39)	1.67 (0.40)	−0.04	0.08	0.650

FT = full-term; PT = preterm; PSI = Parenting Stress Index.

**Table 3:** Spearman’s intercorrelations between mind-mindedness and the parenting stress of mothers participating in the study.

	AMRC	NAMRC	PSI PD	PSI CDI	PSI DC
AMRC	-				
NAMRC	0.02	-			
PSI PD	0.14	0.01	-		
PSI CDI	0.03	0.08	0.52 **	-	
PSI DC	0.14	0.24 *	0.46 **	0.54 **	-
PSI TOT	0.12	0.13	0.86 **	0.77 **	0.77 **

FT = full-term; PT = preterm; AMRC = appropriate mind-related comments; NAMRC = non-attuned mind-related comments; PSI PD = Parenting Distress; PSI CDI = Child Difficult Interaction; PSI DC = Difficult Child; PSI TOT = Total Stress. * *p* < 0.05, ** *p* < 0.001.

**Table 4:** Spearman’s intercorrelations between mind-mindedness and the parenting stress of the PT and FT mothers participating in the study.

		PSI PD	PSI CDI	PSI DC	PSI TOT
AMRC	FT	0.09	0.07	0.09	0.14
	PT	0.22	−0.07	0.18	0.12
NAMRC	FT	0.06	−0.01	0.22	0.14
	PT	−0.05	0.17	0.27 *	0.12

FT = full-term; PT = preterm; AMRC = appropriate mind-related comments; NAMRC = non-attuned mind-related comments; PSI PD = Parenting Distress; PSI CDI = Child Difficult Interaction; PSI DC = Difficult Child; PSI TOT = Total Stress. * *p* < 0.05.
